# Does bathymetry drive coastal whale shark (*Rhincodon typus)* aggregations?

**DOI:** 10.7717/peerj.4904

**Published:** 2018-06-08

**Authors:** Joshua P. Copping, Bryce D. Stewart, Colin J. McClean, James Hancock, Richard Rees

**Affiliations:** 1 School of Environment and Life Sciences, University of Salford, Manchester, United Kingdom; 2 Environment Department, University of York, York, United Kingdom; 3 Maldives Whale Shark Research Programme, York, United Kingdom

**Keywords:** Whale shark, Marine megafauna, Conservation, Bathymetry, Distribution model

## Abstract

**Background:**

The whale shark (*Rhincodon typus*) is known to aggregate in a number of coastal locations globally, however what causes these aggregations to form where they do is largely unknown. This study examines whether bathymetry is an important driver of coastal aggregation locations for *R. typus* through bathymetry’s effect on primary productivity and prey availability. This is a global study taking into account all coastal areas within *R. typus’* range.

**Methods:**

*R. typus* aggregation locations were identified through an extensive literature review. Global bathymetric data were compared at *R. typus* aggregation locations and a large random selection of non-aggregation areas. Generalised linear models were used to assess which bathymetric characteristic had the biggest influence on aggregation presence.

**Results:**

Aggregation sites were significantly shallower than non-aggregation sites and in closer proximity to deep water (the mesopelagic zone) by two orders of magnitude. Slope at aggregation sites was significantly steeper than non-aggregation sites. These three bathymetric variables were shown to have the biggest association with aggregation sites, with up to 88% of deviation explained by the GLMs.

**Discussion:**

The three key bathymetric characteristics similar at the aggregation sites are known to induce upwelling events, increase primary productivity and consequently attract numerous other filter feeding species. The location of aggregation sites in these key areas can be attributed to this increased prey availability, thought to be the main reason *R. typus* aggregations occur, extensively outlined in the literature. The proximity of aggregations to shallow areas such as reefs could also be an important factor why whale sharks thermoregulate after deep dives to feed. These findings increase our understanding of whale shark behaviour and may help guide the identification and conservation of further aggregation sites.

## Introduction

Marine ecosystems are the most extensive and among the most threatened in the world (MEA, 2005). The increasing rates of exploitation and incidental capture in the fisheries industry (Halpern et al., 2007) often exhibit disproportionate impacts on marine megafauna (Abercrombie, Balchowsky & Paine, 2005; Read, Drinker & Northridge, 2006; Capietto et al., 2014). The consequences and subsequent declines are far reaching; marine megafauna are often apex predators, therefore, their removal has cascading impacts on lower tropic levels, causing community restructuring and enhanced vulnerability of other species (Lewison et al., 2004; Casini et al., 2008; Howarth et al., 2014). Impacts are not only ecological; presence of megafauna often significantly contributes to local economies through ecotourism (Catlin & Jones, 2010). Despite their importance, little is understood about the factors affecting the movements and global distributions of many marine megafauna species. Identifying and understanding areas used in important periods of their life is essential for future conservation efforts (Hays et al., 2016). The whale shark (*Rhincodon typus*) is one such species where there are still many knowledge gaps about their global distribution, movements and the factors affecting these (Sequeira et al., 2013).

*Rhincodon typus* is one of three large pelagic filter feeding shark species and is the largest fish in the world (Smith, 1828). They have a circumglobal distribution, found in tropical and warm temperate seas between the latitudes of 30°N and 35°S (Pierce & Norman, 2016). As filter feeders, *R. typus* primarily feed on zooplankton and therefore tend to be observed in areas with high productivity (Colman, 1997; Rohner et al., 2018). However, recent studies have shown whale sharks also feed on the spawn of various corals, fish and invertebrates (Graham & Roberts, 2007; Hobbs et al., 2009; Rohner et al., 2016). There is no robust population estimation for this species, but it is listed as endangered on the IUCN Red List (Pierce & Norman, 2016). Their late sexual maturation, highly mobile nature and relatively low abundance make this species extremely vulnerable, particularly to incidental capture and overexploitation, which has caused global population decline and fragmentation (Dulvy et al., 2008; Pierce & Norman, 2016). More recently, the economic value of live whale sharks to ecotourism has been shown to be considerably higher than when fished (Catlin & Jones, 2010; Cagua et al., 2014). A report published by the WWF-Philippines (Pine, 2007) estimates the value of a whale shark to be worth US$250,000 dead, or US$2,000,000 provided in ecotourism benefits over the animal’s lifetime.

The importance of this species has triggered a growing interest amongst the scientific community, with the majority of papers on *R. typus* published within the past two decades. Together with the increases in ecotourism, there is now a depth of knowledge about local and regional whale shark ecology and biology. Yet there is still a dearth of information about global distribution, especially connectivity of populations and pattern in movements (Sequeira et al., 2012; Sequeira et al., 2013). *R. typus* were once perceived to be solitary animals that live and feed in the open ocean, but it is now well documented that juvenile whale sharks do aggregate in response to the forming of predictable, seasonal feeding opportunities (Colman, 1997; Heyman et al., 2001; Riley et al., 2010).

An increasing number of coastal aggregation areas are being discovered, with research showing there could be up to 25 sites globally (Colman, 1997; Eckert et al., 2002; Andrzejaczek et al., 2016). Nevertheless, 25 sites is a very small number in all of the world’s oceans, indicating that whale sharks only aggregate in response to a very specific set of conditions. There is great variability among aggregation events in terms of available prey, when they occur and the number of individuals aggregating (Rowat & Brooks, 2012; Andrzejaczek et al., 2016). Studies have shown *R. typus* aggregate in areas of high biological productivity and the seasonal nature of such aggregations appears to be the result of local increases in prey (Meekan et al., 2006; Rowat et al., 2007; Rowat et al., 2009). The timing of a number of aggregations is predictable due to the close association with increased prey availability. For example, Heyman et al. (2001) show whale sharks are only regularly seen in large numbers at Gladden Spit, Belize, during full moon periods in April and May where a large number aggregate to feed on Lutjanidae spp. spawn. This is again reflected in the Gulf of Tadjouran, Djibouti, when throughout the winter months (November to February) a large aggregation of *R. typus* can be observed feeding on zooplankton (Rowat et al., 2007). There are disparities in aggregation size and structure between sites; numbers vary from 10 to 1,000-plus individuals and exhibit a spatial segregation by age and gender, typical in shark populations (Springer, 1967; Brunnschweiler et al., 2009; De la Parra Venegas et al., 2011).

*R. typus* is a pelagic species believed to spend most of their lives in deep offshore waters except during aggregation events, which are thought to be predominantly seasonal (Abercrombie, Balchowsky & Paine, 2005; Andrzejaczek et al., 2016). There have only been a handful of studies researching the deep diving behaviour of *R. typus*, nonetheless they suggest *R. typus* dive to the mesopelagic zone to feed (Graham, Roberts & Smart, 2006; Brunnschweiler et al., 2009; Sun et al., 2016). Using pop-up satellite archival tags, Tyminski et al. (2015) recorded one individual diving to a depth of 1,928 m in the north-eastern Gulf of Mexico. These studies indicate deep water is important for *R. typus*, therefore the neighbouring bathymetry could play a role in aggregation events.

Bathymetric features such as continental and reef slopes, shallow banks and seamounts tend to be areas of high marine productivity, in particular high zooplankton abundance, often driving predator prey aggregations (Afonso, McGinty & Machete, 2014). Bouchet et al. (2015) show areas with complex bathymetry such as seamounts or steep slopes found on outer reefs accumulate zooplankton, which subsequently attracts filter feeders, particularly at epipelagic and mesopelagic depths. This was shown by Sims (2008) with a greater abundance of basking sharks (*Cetorhinus maximus*) in areas with steeper slopes, owing to the higher densities of zooplankton.

Studies have shown almost all *R. typus* aggregations occur in coastal areas of shallow bathymetry in close proximity to the reef slope and deeper water (De la Parra Venegas et al., 2011; Donati et al., 2016; Diamant et al., 2016). Past studies have illustrated increased zooplankton availability in areas of steep bathymetry, which *R. typus* preys upon (Bouchet et al., 2015). Therefore, bathymetry at and around areas of aggregation events could be an important factor in driving them. There are currently few in-depth studies into bathymetry and *R. typus* globally (Sleeman et al., 2007; Rohner et al., 2013; Sequeira et al., 2012) use coarse scale bathymetry data to look at the distribution of *R. typus*, and McKinney et al. (2012) use bathymetry to investigate the feeding habitat of *R. typus* in the Gulf of Mexico. However, the current lack of highly detailed studies into bathymetry and *R. typus* could limit future conservation efforts for this species (Rowat & Brooks, 2012).

Our study therefore aims to address this knowledge gap by investigating whether there is an association between bathymetry and *R. typus* feeding aggregation sites by quantitatively analysing previous qualitative and anecdotal observations. We will address the following questions: Are there similarities in bathymetry between aggregation sites? How does this compare to bathymetry at non-aggregation sites? Which bathymetric variables are most associated with aggregations? And, what is the biggest potential driver of aggregations? By addressing these key questions, we will deliver a quantitative update to previous qualitative research and aim to provide an increased understanding of the bathymetric conditions at *R. typus* aggregation sites.

## Materials & Methods

### Data acquisition

To identify *R. typus* aggregation sites, an extensive literature review was done using the following search terms in Web of Science and Google Scholar: Whale shark, *Rhincodon typus*, aggregation, coastal, bathymetry, topography, relief, depth, movements, feeding. All relevant articles (in excess of 150) were evaluated and papers that mentioned aggregations were retained for further use. From these, a database of 17 aggregation events was created containing size, spatial and temporal occurrence and coordinates at the centre of the aggregation. Although more sites were found in the literature search (Gulf of Oman (Robinson et al., 2016), Japan (Colman, 1997), Tawian (Stewart & Wilson, 2005; Hsu, Joung & Liu, 2012) and Thailand (Theberge & Dearden, 2006)), there was a lack of detailed location information and no clarity as to if these were recognised aggregations or one-off events. Therefore, these potential additional sites could not be used accurately in our analysis and were not included in this study. See Table 1 for a list of the included aggregation sites.

**Table 1:** Summary of the aggregation sites. Names and locations used in this study with the literature sources where information has been extracted.

Aggregation location	Sources
Australia, Ningaloo Reef	Meekan et al. (2006), Wilson et al. (2006), Reynolds et al. (2016) and Norman et al. (2017)
Belize, Gladden Spit	Heyman et al. (2001), Graham, Roberts & Smart (2006), Quiros (2007) and McKinney et al. (2017)
^a^Christmas Island, West Coast	Hobbs et al. (2009) and Meekan et al. (2009)
Djibouti, Gulf of Tadjoura	Rowat et al. (2007), Schmidt et al. (2009), Rowat & Brooks (2012) and Leblond & Rowat (2016)
Gulf of California, La Paz	Clark & Nelson (1997), Ramírez-Macías et al. (2016) and Ramírez-Macías & Saad (2016)
^a^Gulf of Mexico, North Area	Hoffmayer et al. (2007) and McKinney et al. (2012)
India, Gujarat	Pravin (2000), Rowat (2007) and Sequeira et al. (2014)
Madagascar, Nosy Be	Jonahson & Harding (2007), Brunnschweiler et al. (2009) and Diamant et al. (2016)
Maldives, South Ari Atoll	Riley et al. (2010), Donati et al. (2016) and Perry (2017)
Mexico, Afuera	De la Parra Venegas et al. (2011), Hueter, Tyminski & De la Parra (2013) and Hacohen-Domené et al. (2015)
Mexico, Yucatan Peninsula	Motta et al. (2010), Ziegler, Dearden & Rollins (2012), Hueter, Tyminski & De la Parra (2013) and Tyminski et al. (2015)
Mozambique, Tofo Beach	Brunnschweiler et al. (2009), Sequeira et al. (2012) and Rohner et al. (2018)
Philippines, Donsol Bay	Eckert et al. (2002), Quiros (2007), Thomson et al. (2017) and Araujo et al. (2017)
^a^Qatar, Al-Shaheen Oil Field	Robinson et al. (2013), Robinson et al. (2016) and Robinson et al. (2017)
Seychelles,	Rowat et al. (2007), Rowat et al. (2009) and Andrzejaczek et al. (2016)
Saudi Arabia, Al-Lith	De la Torre et al. (2012), Berumen et al. (2014) and Sun et al. (2016)
Tanzania, Mafia Island	Cochran (2014), Rohner et al. (2015) and Prebble et al. (2016)

**Notes.**

a indicate they were later removed from analysis, explained in subsequent sections.

Furthermore, three of the 17 aggregation sites appeared vastly different from the other sites in this study. The Al Shaheen Oil Field site aggregation, Qatar, described by Robinson et al. (2013) is an area of high whale shark abundance, however it is not coastal and is dispersed over a large area, therefore not considered a truly comparable aggregation. Literature suggests the aggregations around the Mississippi Delta and the Christmas Island aggregation are driven by unique ecological phenomena. The aggregation in the Gulf of Mexico appears to be driven by runoff from the Mississippi stimulating high primary productivity at a scale far higher than other aggregation sites (Hoffmayer et al., 2007; Hueter, Tyminski & De la Parra, 2013). Meanwhile, the Christmas Island aggregation is driven by red crab (*Gecarcoidea natalis*) spawning events which do not occur at any other sites (Hobbs et al., 2009; Meekan et al., 2009). Moreover, these three sites have considerably fewer literature describing the aggregations compared to the other sites in this study. Therefore, for the purpose of this study we decided these sites were not comparable with the other 14 well-known large aggregations.

The global range of *R. typus* was obtained from the IUCN (International Union for Conservation of Nature , IUCN) to examine the spatial distribution of aggregation sites. Bathymetric depth data were obtained from the 2014 General Bathymetric Chart of the Oceans (GEBCO, 2015) at a resolution of 30 arc-seconds (approximately 1 km). The GEBCO dataset was highest resolution, freely available bathymetric that covered the whole study area. Whilst more detailed datasets are available for specific areas such as Western Australia, there is no comparable data for many of the areas in the tropics, therefore the GEBCO dataset was used throughout to ensure consistency and to allow comparison between sites. To examine relationships between bathymetry, primary productivity and their association with whale shark aggregations we also obtained data on sea surface temperature (SST) and chlorophyll-a concentration from OceanColor (NASA, 2014), an archive of oceanographic data from satellite based remote sensing. SST data were a seasonal composite of the years 2000 to 2016 at a 4 km resolution recorded by the Terra MODIS instrument. The seasonal composite of chlorophyll-a concentration from 2012 to 2016 at a 4 km resolution, recorded by the SNPP-VIIRS instrument was also used. Seasonal composites were used as *R. typus* aggregate seasonally and a composite of a number of years should mitigate the influence of anomalies such as El Niño.

### Spatial analysis

**Figure 1:**
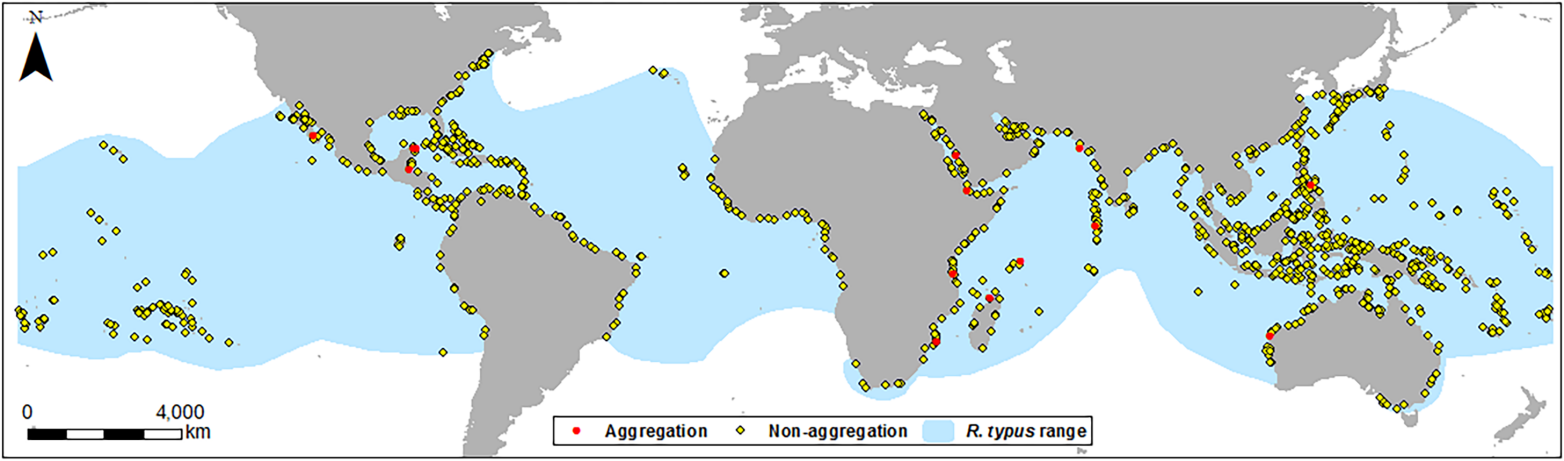
Location map of sites. Location of aggregation and non-aggregation sites used in this study with the global *R. typus* distribution.

*R. typus* aggregation point data were imported, and locations re-checked against source papers to ensure locations were correct. To investigate if aggregation sites are unique, they were compared to areas where aggregations do not occur; therefore, 1,000 random points in non-aggregation areas were created (Fig. 1). 1,000 points were chosen to allow a large geographic spread within *R. typus’* range to capture a range of bathymetric characteristics. Whilst there is no consensus on the correct number of pseudo absence points to include, the large number of pseudo absence points we used allowed for a powerful comparison with aggregation sites. Although *R. typus* has a global range, the aggregations in this study are coastal, therefore unproductive and deep high seas areas were excluded, as these would have biased our absence data. Thus, random points were constrained to coastal areas by calculating the maximum distance of the observed aggregation sites from the coast (26 km) and using this to create a zone around coastlines within *R. typus’* range. Due to the resolution of the bathymetry data, each point covers an area of approximately 1 km^2^. Aspect, slope and vertical/profile curvature, hereafter referred to as “curvature”, layers were generated from bathymetric depth to further examine the characteristics of bathymetry in key areas. Positive values of curvature indicate upwardly concave slopes, negative values show upwardly convex slopes, and values close to 0 indicate planar slopes. Benthic complexity was generated from the standard deviation of depth and slope roughness was generated as the standard deviation of slope. Although there are other ways to measure complexity and roughness, using the standard deviation of slope or depth is one of the most simple and effective measures at a variety of scales (Fox & Hayes, 1985; Grohmann, Smith & Riccomini, 2011). These variables were chosen based on similar research focusing on both *R. typus* and other marine megafauna species (Sleeman et al., 2007; Sequeira et al., 2012; Afonso, McGinty & Machete, 2014; Bouchet et al., 2015).

Twenty km buffer zones were generated around each aggregation and random point, allowing extraction of information about the surrounding areas. This size of zone was selected based on distances *R. typus* travels; Hueter, Tyminski & De la Parra (2013) show the mean daily distance travelled during long distance movements to be 24.7 km, which would be lower during aggregation events with the focus on feeding. Minimum, maximum, mean, range were extracted for depth, complexity, slope and curvature for the point data and buffer zones. SST and chlorophyll data were extracted by the same method but only for point data, not buffer zones due to the lower resolution of these data, where one cell covers a large portion of the buffer zone. The distance of aggregation sites to the 200 m and 1,000 m isobaths (mesopelagic and bathypelagic zones) were also measured as these depths were previously shown to be important for *R. typus* feeding (Brunnschweiler et al., 2009).

### Statistical analysis

Of the 1,000 random points, five had missing data for one or more variables so were removed, leaving 995 random points for analysis. Literature shows aggregations occur in the seasons with the highest chlorophyll-a concentration and SST (Sequeira et al., 2012; Afonso, McGinty & Machete, 2014). Therefore, maximum values across the four seasons were extracted and used during analysis. Basic statistics were run on the variables, examining means and variance to initially look for differences in the data. To test whether differences between aggregation and non-aggregation sites were significant, an independent samples *t*-test was used. This was chosen due to its power and robustness, particularly with large datasets (Zimmerman, 1987; Erceg-Hurn & Mirosevich, 2008). Generalised linear models (GLMs) with a binomial error function were used to investigate which variable had the greatest influence on aggregation presence. Whilst examining data, a number of predictor variables exhibited collinearity (Variance Inflation Factors ≤5.0 or Pearsons Correlation *r* ≥ 0.7). Variables with the strongest covariate relationship were removed from the dataset, strongest first, in an iterative stepwise process, whilst retaining the variables with the strongest univariate relationship with the response variable. To minimise this intercorrelation and prevent masking of trends whilst modelling, data were split into four sub-sets based on the stepwise reduction (Zuur, Ieno & Elphick, 2010; Tamura et al., 2017). These four sub-sets were modelled separately to keep variables with strong covariate relationships apart (see Table 2 for the list of variables in each GLM). The four model outputs showed no overdispersion so minimum adequate models were created using backward-forward stepwise reduction based on the Akaike Information Criterion (AIC) (Hilbe, 1994; Dobson & Barnett, 2008). Analysis of deviance was used to test whether the deviance explained had not reduced from the full models, therefore justifying the use of stepwise reduction (McCulloch, 2000).

**Table 2:** Summary of the variables used in this study. These have been extracted from spatial analysis and the name they are referred to throughout this study.

Variable	Name
Mean point depth	Site depth
Mean buffer zone depth	Buffer depth
Maximum buffer zone depth	Buffer max
Buffer zone depth range	Buffer range
Benthic complexity	Complexity
Mean point slope	Site slope
Mean buffer zone slope	Buffer slope
Maximum buffer zone slope	Max slope
Slope roughness	Roughness
Curvature at point	Site curvature
Mean curvature in buffer zone	Mean curvature
Maximum curvature in buffer zone	Concavity
Minimum curvature in buffer zone	Convexity
Slope aspect	Aspect
Proximity to 200 m isobath	200 m
Proximity to 1,000 m isobath	1,000 m
Mean Chlorophyll-a concentration	Chlorophyll
Mean SST	SST

## Results

Many of the bathymetric variables studied showed distinct differences between the aggregation and non-aggregation sites. All depth measures were significantly shallower in areas *R. typus* aggregate (Table 3), with the biggest difference shown to be between mean depth at the aggregation sites, 22.2 m, an order of magnitude lower than non-aggregation sites (635.3 m); this difference was highly significant (*t* = 19.262, *df* = 1006.7, *p* < 0.001). Similarly, the mean depth in 20 km buffer zones was shown to be significantly shallower than mean depth in non-aggregation sites (*t* = 9.578, *df* = 25.601, *p* < 0.001). Maximum depth and range of depths within the 20 km buffer zones were around 50% lower at aggregation sites, further highlighting aggregations occur in shallower areas. Benthic complexity was significantly lower at aggregation sites than that at non-aggregation sites (Table 3), meaning a smoother, less rugged surface.

**Table 3:** Means for each variable and results of the *t*-tests. Means of each variable at aggregation and non-aggregation sites with *t*-test statistic results highlighting the difference.

Variable	Aggregation site mean	Non-aggregation site mean	*t*-test statistics
Site depth (m)	22.2	635.3	*t* = 19.262, *df* = 1006.7, *p* < 0.001
Buffer depth (m)	173.0	713.6	*t* = 9.578, *df* = 25.601, *p* < 0.001
Buffer max (m)	716.6	1,350.8	*t* = 3.086, *df* = 14.441, *p* = 0.008807
Buffer range (m)	712.4	1,295.3	*t* = 2.836, *df* = 14.319, *p* = 0.013
Complexity	180.3	336.5	*t* = 2.571, *df* = 14.121, *p* = 0.022
Site slope (degrees)	0.67780	0.00003	*t* = 2.931, *df* = 13, *p* = 0.012
Buffer slope (degrees)	1.41	2.55	*t* = 2.951, *df* = 14.46, *p* = 0.011
Max slope (degrees)	8.95	10.31	*t* = 0.624, *df* = 13.616, *p* = 0.543
Roughness	0.00744	1.96266	*t* = 30.083, *df* = 994, *p* < 0.001
Site curvature	0.000002	0.000011	*t* = 1.274, *df* = 21.855, *p* = 0.216
Mean curvature	0.000005	0.000003	*t* = 1.386, *df* = 13.177, *p* = 0.189
Concavity	0.000613	0.000584	*t* = 0.181, *df* = 13.549, *p* = 0.859
Convexity	−0.000648	−0.000594	*t* = 0.312, *df* = 13.444, *p* = 0.759
200 m (km)	0.170	71.781	*t* = 14.477, *df* = 994.2, *p* < 0.001
1,000 m (km)	0.416	99.385	*t* = 17.694, *df* = 994.32, *p* < 0.001
Aspect	190.498	172.826	*t* = 0.73397, *df* = 13.518, *p* = 0.475
Chlorophyll (mg/m^3^)	0.669	1.532	*t* = 6.397, *df* = 35.86, *p* < 0.001
SST (°C)	30.4	29.8	*t* = 2.173, *df* = 18.129, *p* = 0.063

The mean slope at aggregation sites was significantly steeper than that at non-aggregation sites, however the inverse was displayed with mean slope in the buffer zones (Table 3). *T*-tests showed no significant differences between the means of maximum slope in buffer zones, with a difference of only 1.4 degrees between aggregation and non-aggregation sites. However, the absolute maximum slope recorded was steeper; 29.8 degrees at aggregation sites and 60.1 degrees at non-aggregation sites. Aggregation sites had a lower slope roughness by three orders of magnitude compared to non-aggregation sites, and the t-tests showed these differences were highly significant. The curvature at and around both aggregation and non-aggregation sites was slightly concave with no real differences (Table 3). The buffer zones around aggregation sites showed greater concavity than areas around non-aggregation sites and the result is the same when looking at the slight convexity displayed at both sites. With such small variation in all mean values, there were no significant differences between curvature at aggregation and non-aggregation sites.

Aggregation sites were two orders of magnitude closer to the 200 m isobaths with a mean distance of 0.14 km, whereas the mean distance for non-aggregation sites was 71.78 km. Similar results are shown in the distance to the 1,000 m isobaths; aggregation sites had a mean distance of 0.41 km compared to non-aggregation sites which had a mean distance of 99.38. The observed differences in distance to both the 200 m and 1,000 m isobaths were highly significant (Table 3). The mean slope aspect was south for both aggregation and non-aggregation sites; the majority of individual slope aspects were either southeast, south or southwest, with very few facing north. There were differences in chlorophyll-a concentration and SST between aggregation and non-aggregation sites. Surprisingly, chlorophyll-a was significantly lower at aggregation sites with a mean concentration of 0.67 mg/m^3^, compared to 1.53 mg/m^3^ at non-aggregation sites (*t* = 6.397, *df* = 35.86, *p* < 0.001). SST showed no significant difference between aggregation sites, and non-aggregation sites.

### Main drivers of aggregations

The six predictor variables left in the four minimum adequate models were significant (*p* < 0.05) and three of the models (Table 4) had high percentage of deviance explained (>85%) for aggregation site presence. Aggregation site presence was best modelled by GLM3, with the mean depth in the buffer zone (buffer depth) explaining 88.71% of deviance. GLM1 containing mean depth at points (site depth) and proximity to the 200 m isobaths explained 87.96% of deviance of aggregation sites. Site slope and proximity to 1,000 m, which were left in GLM2 after stepwise reduction, explained 88.16% of aggregation site deviance. These three GLMs all explain high deviation with significance, therefore indicating commonality in bathymetric features at aggregation sites and differences to those found at non-aggregation sites. Diagnostic plots were checked for outliers and showed the residuals were close to the line and Cook’s Distance values below 0.5 for all points, suggesting no single point had an overpowering or unnecessary influence on the overall trend of aggregation site presence. *P*[*D*] values (probability of decreased deviance explained from the full model) for all models were high, suggesting the minimum adequate models used explain no less deviance than the full GLMs and the stepwise reduction of variables was justified.

**Table 4:** Results of the GLM. Binomial generalised linear models of aggregation site presence and absence bathymetric and environmental predictor variables. Statistics include the percentage deviance explained (%D), probability of deviation (p[t]) and the probability of decreased deviance explained form the full model (p[D]). Bold variables indicate significance to a level of 0.05.

Model Name	Predictor variables tested	Minimum adequate model
GLM1	**Site depth**, **200 m**, Site curvature, Complexity	**Site depth:**%*D* = **0.7748**, ***p[t]*** = **0.0143**, **200 m:**%*D* = **0.7496**, ***p[t]*** = **0.0379**, (AIC = 23.2, %*D* = 0.8796, *p*[*D*] = 0.683)
GLM2	**Site slope**, Depth range, **1,000 m**, Mean curvature, Max slope	**Site gradient:**%*D* = **0.6571**, ***p[t]*** = **0.0021**, **1,000 m:**%*D* = **0.4885**, ***p[t]*** = **0.0157**, (AIC = 27.3, %*D* = 0.8816, *p*[*D*] = 0.784)
GLM3	**Buffer depth**, Roughness, SST, Convexity, Concavity	**Buffer depth:**%*D* = **0.8871**, ***p[t]*** = **0.029**, (AIC = 19.4, %*D* = 0.8871, *p*[*D*] = 0.793
GLM4	Buffer slope, **Buffer max**, Aspect, Chlorophyll	**Buffer max:**%*D* = **0.4631**, ***p[t]*** = **0.0011**, (AIC = 67.006, %*D* = 0.4631, *p*[*D*] = 0.538)

## Discussion

Our analysis shows bathymetry to be significantly different at and around coastal areas where *R. typus* aggregate compared to coastal areas where aggregations do not occur. Aggregation sites were significantly shallower over both spatial scales (point data and the 20 km buffer zone). Despite the area surrounding aggregation sites being roughly 500 m shallower than that measured at non-aggregation sites, the mean distance of aggregation sites to both the mesopelagic and bathypelagic zones was significantly closer by two orders of magnitude than non-aggregation sites. Steeper slopes were found at the aggregation sites, but not in the surrounding buffer zones. These results suggest three aspects of bathymetry are important to aggregation formation; shallow areas at aggregation sites, proximity to deep water and steep slopes. These bathymetric characteristics were found to be important in a number of local and regional studies of *R. typus* distribution (McKinney et al., 2012; Sequeira et al., 2012; Afonso, McGinty & Machete, 2014).

Site depth and buffer zone depth were among the biggest drivers of aggregations. Literature extensively illustrates *R. typus* aggregating in shallow water for two main reasons. Firstly, primarily to feed, hence chlorophyll-a concentration being included in this study as an indicator of planktonic productivity (Platt & Herman, 1983; Sequeira et al., 2012). However, non-aggregation sites had a mean chlorophyll-a concentration twice as high as that at aggregation sites. The mean chlorophyll-a concentration at aggregation sites of 0.67 mg/m^3^ can be considered relatively high compared to pelagic areas where typically chlorophyll-a concentration is <0.25 mg/m^3^ (Hu, Lee & Franz, 2012), nevertheless it was significantly lower than other coastal areas within *R. typus*’ range. One reason for this can be attributed to the diversity of *R. typus’* prey; a number of studies have shown aggregations coincide with spawning events such as Scombridae spp. spawn at the Yucatan peninsula (De la Parra Venegas et al., 2011) or Lutjanidae spp. spawn in Belize (Heyman et al., 2001). However, Donati et al. (2016) describe the aggregation at South Ari atoll as a yearlong phenomenon with no seasonal peak and there is a current lack of understanding as to the main cause of this aggregation if it is not seasonally prey driven. Nonetheless, these whale sharks will feed on plankton and various spawn in the shallow coastal waters where they are regularly observed (Hoffmayer et al., 2007; Motta et al., 2010).

The second main reason suggested for aggregations in shallow waters is for thermoregulation after deep dives into cooler water. Research into this field is limited (Brunnschweiler et al., 2009; Thums et al., 2013; Tyminski et al., 2015), however this is a viable theory and the results of this study illustrate aggregations occurred in warmer waters than the random non-aggregation sites selected. Although SST was only higher by ∼0.5 °C, this was at a coarse resolution, larger than the aggregation areas, therefore SSTs on site may in fact be higher. Furthermore, the temporal averaging of SSTs may have influenced the extent of difference between sites (this was done to ensure consistency and avoid anomalous data produced by El Niño years). A number of ectothermic species require surface intervals to raise body temperature to levels needed to regulate physiological processes after time spent foraging in cooler, deep waters (Thums et al., 2013). The size of *R. typus*’ gills make them extremely efficient at filtering prey from the water, but the large volume of water passing over the gills causes *R. typus* to cool relatively quickly when in deeper water (Colman, 1997). If thermoregulation occurs in warm shallow areas with high productivity, or an abundance of prey, *R. typus* could continue to feed whilst increasing body temperature from deep dives.

The proximity of *R. typus* aggregations to deep water is therefore thought to be due to frequent deep dives for prey (Graham, Roberts & Smart, 2006; Tyminski et al., 2015), whilst remaining close to shallow areas of high productivity for thermoregulation (Thums et al., 2013) and potential feeding. All aggregation sites in this study were significantly closer to areas with water in the mesopelagic and bathypelagic zones. The deep water bathymetric variables (site depth, buffer depth and proximity to the 200 m and 1,000 m isobaths) explained the greatest deviance of *R. typus* aggregation site presence, showing these aspects may be highly important for separating aggregation from non-aggregation sites. Sequeira et al. (2012) found similar results when modelling *R. typus* habitat suitability in the Indian Ocean; depth and distance to the continental shelf were two of the biggest indicators of habitat preference.

A number of studies with tagged whale sharks show their deep diving behaviour; Rowat & Gore (2007) recorded three *R. typus* individuals spent ∼30% of their time at depths of 750–1,000 m. A recent study by Tyminski et al. (2015) showed one individual diving as deep as 1,928 m in temperatures 4.2 °C, similarly reflected by Brunnschweiler et al. (2009) in which two tagged whale sharks were recorded at depths of 1,286 m in temperatures of 3.4 °C. Graham, Roberts & Smart (2006) carried out a similar study, further illustrating deep diving behaviour and also recording available prey at these depths. It has been suggested *R. typus* feeds on zooplankton (euphausiids and myctophids), squid and jellyfish in these deep waters seaward of the shelf breaks (Graham, Roberts & Smart, 2006; Wilson et al., 2006). Although chlorophyll-a concentration was lower at aggregation sites, the satellite sensors cannot penetrate deeper than 60 m (Mélin & Hoepffner, 2011), causing deep-water areas with high productivity, such as around slopes and shelfs, to be missed. These areas have been shown to be highly important to *R. typus* for feeding in a number of studies (Graham, Roberts & Smart, 2006), and more recently Sequeira et al. (2012) and McKinney et al. (2012) showed areas with these bathymetric characteristics are the most associated with *R. typus* sightings.

Similarly, the basking shark (*Cetorhinus maximus*) and megamouth shark (*Megachasma pelagios*) have also been recorded diving into the mesopelagic and bathypelagic zones in search of prey (Nelson et al., 1997; Sims et al., 2003; Gore et al., 2008). Wilson et al. (2006) hypothesise the deep diving behaviour in all three species is to locate the deep scattering layer and associated prey at dusk and dawn. This idea was further supported by Gore et al. (2008) who suggested regular dives of increasing depths is indicative of systematic foraging, supporting the theory that deep dives occur to locate horizontally dispersed prey. However, despite research into deep diving of these shark species, the function of deep dives for *R. typus* remains poorly understood.

In this study, *R. typus* aggregations occurred in relatively flat areas with a mean slope of 0.68 degrees, however, this was significantly steeper than non-aggregation sites. Steepness of slopes increased further from aggregation sites with a mean of 1.4 degrees and absolute maximum of 29.84 degrees in the 20 km buffer zone. Aggregations typically occur in the fore reef and lagoon areas, leading out to the reef slope, reef wall or continental slope, which has a steeper slope and deeper water.

Areas with steep slopes are known to induce upwelling events (Botsford et al., 2003; Zavala-Hidalgo et al., 2006), particularly coastal areas where depth changes rapidly, forcing offshore deep-water currents to deflect against the steep slopes, bringing nutrient rich water to the surface (Jacox & Edwards, 2011; Connolly, 2013). These areas have biological significance, and often associated with enhanced primary productivity, therefore increasing plankton abundance and attracting a number of species throughout the trophic levels (Botsford et al., 2003; Jacox & Edwards, 2011).

Wolanski & Hamner (1988) carried out one of the first studies on the biological impacts of steep bathymetry, suggesting these areas are of great significance to large marine species due to availability of prey. Sims (2008) confirmed this with *Cetorhinus maximus*, as steep slopes were shown to be their most common foraging habitat, where the highest zooplankton densities were observed. McKinney et al. (2012) modelled the feeding habitat of *R. typus* aggregations in the Gulf of Mexico using bathymetry, showing areas close to the continental shelf are often selected as aggregation sites due to their productivity. The model of McKinney et al. (2012) suggested proximity to a continental shelf is one of the biggest influences on aggregation site location. Subsequent research by Afonso, McGinty & Machete (2014) show increased *R. typus* abundance in areas with steep bathymetric slope and in areas associated with increased prey abundance.

Because *R. typus* aggregate in only a handful coastal areas and aggregation events are highly predictable, these sites should be focal points for conservation efforts to protect this species through a number of means such as MPA creation, fishing restrictions, boat speed limits and limited visitor numbers. By showing that these aggregations occur in areas with specific bathymetry, there is the possibility to use species distribution models or habitat models to predict other suitable areas where aggregations may already occur or areas aggregations may shift to with projected anthropogenic climate change. The aggregations investigated in this study represent only one of a number of habitats used in *R. typus’* life cycle, with these aggregations shown to be used predominantly by juvenile males (Heyman et al., 2001; Riley et al., 2010). There may be other types of aggregation occurring in offshore waters, but there is currently little research being undertaken due to the high economic and time requirements for such research.

Aggregations increase vulnerability to capture, boat strikes and overexploitation (Lewison et al., 2004), particularly during crucial periods in their lives, such as feeding events and breeding, and when sharks may be recovering from deep dives. Whilst there is no evidence *R. typus* aggregations are for breeding, there is lack of information regarding the breeding behaviour of this species. Therefore, it is plausible aggregations could also be used for mating, which has been observed in zebra sharks (*Stegostoma fasciatum*) (Dudgeon, Noad & Lanyon, 2008) and is suspected to occur at *Cetorhinus maximus* aggregations (Wilson, 2004). This study and a handful of others (McKinney et al., 2012; Sequeira et al., 2012; Afonso, McGinty & Machete, 2014) have shown there are defined bathymetric characteristics in *R. typus* aggregations. As certain characteristics and features of bathymetry are of great importance to a number of marine megafauna species, more research should be carried out in this field with conservation efforts focusing on areas where species are at their most abundant, but also at their most vulnerable.

## Conclusion

This study shows clear evidence that there are significant differences in bathymetry between the coastal areas where *R. typus* aggregate compared to areas where *R. typus* aggregations do not occur. Aggregations occur in shallow areas in close proximity to a reef slope or shelf break with a steep slope, which leads into water in the mesopelagic and bathypelagic zones. The bathymetric characteristics at and around aggregation sites are all associated with increased productivity and prey availability, which are the main reasons *R. typus* and a number of other species aggregate. Knowing this, future conservation efforts for marine megafauna could look for areas with these key bathymetric characteristics, which have been shown to be present in areas of aggregations for a number of species. This study is the latest addition to that research; we have shown that key bathymetric characteristics are a feature of areas in which whale sharks aggregate.

##  Supplemental Information

10.7717/peerj.4904/supp-1 Supplemental Information 1 Bathymetry data Values for each variable extracted at each aggregation and non-aggregation site.

## Additional Information and Declarations

### Data Availability

The following information was supplied regarding data availability: The raw data are provided in a Supplemental Information 1.

## References

[ref-1] Abercrombie DL, Balchowsky HA, Paine AL (2005). 2002 and 2003 annual summary: large pelagic species.

[ref-2] Afonso P, McGinty N, Machete M (2014). Dynamics of whale shark occurrence at their fringe oceanic habitat. PLOS ONE.

[ref-3] Andrzejaczek S, Meeuwig JJ, Rowat D, Pierce SJ, Davies TK, Fisher R, Meekan MG (2016). Establishing the ecological connectivity of whale shark aggregations across the Indian Ocean–a photo-identification approach.

[ref-4] Araujo G, Snow S, So CL, Labaja J, Murray R, Colucci A, Ponzo A (2017). Population structure, residency patterns and movements of whale sharks in Southern Leyte, Philippines: results from dedicated photo-ID and citizen science. Aquatic Conservation: Marine and Freshwater Ecosystems.

[ref-5] Berumen ML, Braun CD, Cochran JE, Skomal GB, Thorrold SR (2014). Movement patterns of juvenile whale sharks tagged at an aggregation site in the Red Sea. PLOS ONE.

[ref-6] Botsford LW, Lawrence CA, Dever EP, Hastings A, Largier J (2003). Wind strength and biological productivity in upwelling systems: an idealized study. Fisheries Oceanography.

[ref-7] Bouchet PJ, Meeuwig JJ, Kent S, Chandra P, Letessier TB, Jenner CK (2015). Topographic determinants of mobile vertebrate predator hotspots: current knowledge and future directions. Biological Reviews.

[ref-8] Brunnschweiler JM, Baensch H, Pierce SJ, Sims DW (2009). Deep-diving behaviour of a whale shark Rhincodon typus during long-distance movement in the western Indian Ocean. Journal of Fish Biology.

[ref-9] Cagua EF, Collins N, Hancock J, Rees R (2014). Whale shark economics: a valuation of wildlife tourism in South Ari Atoll, Maldives. PeerJ.

[ref-10] Capietto A, Escalle L, Chavance P, Dubroca L, De Molina AD, Murua H, Floch L, Damiano A, Rowat D, Merigot B (2014). Mortality of marine megafauna induced by fisheries: insights from the whale shark, the world’s largest fish. Biological Conservation.

[ref-11] Casini M, Lövgren J, Hjelm J, Cardinale M, Molinero JC, Kornilovs G (2008). Multi-level trophic cascades in a heavily exploited open marine ecosystem. Proceedings of the Royal Society of London B: Biological Sciences.

[ref-12] Catlin J, Jones R (2010). Whale shark tourism at Ningaloo Marine park: a longitudinal study of wildlife tourism. Tourism Management.

[ref-13] Clark E, Nelson DR (1997). Young whale sharks, *Rhincodon typus*, feeding on a copepod bloom near La Paz, Mexico. Environmental Biology of Fishes.

[ref-14] Cochran J (2014). Characterization of novel whale shark aggregations at Shib Habil, Saudi Arabia and Mafia Island, Tanzania. Doctoral dissertation.

[ref-15] Colman JG (1997). A review of the biology and ecology of the whale shark. Journal of Fish Biology.

[ref-16] Connolly T (2013). Slope and shelf processes associated with upwelling in the northern California Current system. Doctoral dissertation.

[ref-17] De la Torre PR, Salama KN, Berumen ML, Lloyd Smith E (2012). The integrated satellite-acoustic telemetry (iSAT) system for tracking marine megafauna.

[ref-18] De la Parra Venegas R, Hueter R, Cano JG, Tyminski J, Remolina JG, Maslanka M, Ormos A, Weigt L, Carlson B, Dove A (2011). An unprecedented aggregation of whale sharks, *Rhincodon typus*, in Mexican coastal waters of the Caribbean Sea. PLOS ONE.

[ref-19] Diamant S, Pierce SJ, Ramírez-Macías D, Heithaus MR, D’Echon AG, D’Echon TG, Kiszka JJ (2016). Preliminary observations on whale sharks in Nosy Be, Madagascar.

[ref-20] Dobson AJ, Barnett A (2008). An introduction to generalized linear models.

[ref-21] Donati G, Rees RG, Hancock JW, Jenkins TK, Shameel I, Hindle K, Zareer I, Childs A, Cagua EF (2016). New insights into the South Ari atoll whale shark, *Rhincodon typus*, aggregation.

[ref-22] Dudgeon CL, Noad MJ, Lanyon JM (2008). Abundance and demography of a seasonal aggregation of zebra sharks Stegostoma fasciatum. Marine Ecology Progress Series.

[ref-23] Dulvy NK, Baum JK, Clarke S, Compagno LJ, Cortes E, Domingo A, Fordham S, Fowler S, Francis MP, Gibson C, Martínez J (2008). You can swim but you can’t hide: the global status and conservation of oceanic pelagic sharks and rays. Aquatic Conservation: Marine and Freshwater Ecosystems.

[ref-24] Eckert SA, Dolar LL, Kooyman GL, Perrin W, Rahman RA (2002). Movements of whale sharks (*Rhincodon typus*) in South-east Asian waters as determined by satellite telemetry. Journal of Zoology.

[ref-25] Erceg-Hurn DM, Mirosevich VM (2008). Modern robust statistical methods: an easy way to maximize the accuracy and power of your research. American Psychologist.

[ref-26] Fox CG, Hayes DE (1985). Quantitative methods for analyzing the roughness of the seafloor. Reviews of Geophysics.

[ref-27] GEBCO (2015). General bathymetric chart of the oceans, version 20141103. http://www.gebco.net.

[ref-28] Gore MA, Rowat D, Hall J, Gell FR, Ormond RF (2008). Transatlantic migration and deep mid-ocean diving by basking shark. Biology Letters.

[ref-29] Graham RT, Roberts CM (2007). Assessing the size, growth rate and structure of a seasonal population of whale sharks (*Rhincodon typus* Smith 1828) using conventional tagging and photo identification. Fisheries Research.

[ref-30] Graham RT, Roberts CM, Smart JC (2006). Diving behaviour of whale sharks in relation to a predictable food pulse. Journal of the Royal Society Interface.

[ref-31] Grohmann CH, Smith MJ, Riccomini C (2011). Multiscale analysis of topographic surface roughness in the Midland Valley, Scotland. IEEE Transactions on Geoscience and Remote Sensing.

[ref-32] Hacohen-Domené A, Martínez-Rincón RO, Galván-Magaña F, Cárdenas-Palomo N, De la Parra-Venegas R, Galván-Pastoriza B, Dove AD (2015). Habitat suitability and environmental factors affecting whale shark (*Rhincodon typus*) aggregations in the Mexican Caribbean. Environmental Biology of Fishes.

[ref-33] Halpern BS, Selkoe KA, Micheli F, Kappel CV (2007). Evaluating and ranking the vulnerability of global marine ecosystems to anthropogenic threats. Conservation Biology.

[ref-34] Hays GC, Ferreira LC, Sequeira AM, Meekan MG, Duarte CM, Bailey H, Bailleul F, Bowen WD, Caley MJ, Costa DP, Eguíluz VM (2016). Key questions in marine megafauna movement ecology. Trends in Ecology & Evolution.

[ref-35] Heyman WD, Graham RT, Kjerfve B, Johannes RE (2001). Whale sharks *Rhincodon typus* aggregate to feed on fish spawn in Belize. Marine Ecology Progress Series.

[ref-36] Hilbe JM (1994). Generalized linear models. The American Statistician.

[ref-37] Hobbs JA, Frisch AJ, Hamanaka T, McDonald CA, Gilligan JJ, Neilson J (2009). Seasonal aggregation of juvenile whale sharks (*Rhincodon typus*) at Christmas Island, Indian Ocean. Coral Reefs.

[ref-38] Hoffmayer ER, Franks JS, Driggers III WB, Oswald KJ, Quattro JM (2007). Observations of a feeding aggregation of whale sharks, *Rhincodon typus*, in the north central Gulf of Mexico. Gulf and Caribbean Research.

[ref-39] Howarth LM, Roberts CM, Thurstan RH, Stewart BD (2014). The unintended consequences of simplifying the sea: making the case for complexity. Fish and Fisheries.

[ref-40] Hsu HH, Joung SJ, Liu KM (2012). Fisheries, management and conservation of the whale shark *Rhincodon typus* in Taiwan. Journal of Fish Biology.

[ref-41] Hu C, Lee Z, Franz B (2012). Chlorophyll *a* algorithms for oligotrophic oceans: a novel approach based on three-band reflectance difference. Journal of Geophysical Research.

[ref-42] Hueter RE, Tyminski JP, De la Parra R (2013). Horizontal movements, migration patterns, and population structure of whale sharks in the Gulf of Mexico and northwestern Caribbean Sea. PLOS ONE.

[ref-43] Jacox MG, Edwards CA (2011). Effects of stratification and shelf slope on nutrient supply in coastal upwelling regions. Journal of Geophysical Research: Oceans.

[ref-44] Jonahson M, Harding S (2007). Occurrence of whale sharks (*Rhincodon typus*) in Madagascar. Fisheries Research.

[ref-45] Leblond ST, Rowat DR (2016). Studying spatial distribution of the whale shark in the Gulf of Tadjora, Djibouti.

[ref-46] Lewison RL, Crowder LB, Read AJ, Freeman SA (2004). Understanding impacts of fisheries bycatch on marine megafauna. Trends in Ecology & Evolution.

[ref-47] McCulloch CE (2000). Generalized linear models. Journal of the American Statistical Association.

[ref-48] McKinney JA, Hoffmayer ER, Holmberg J, Graham RT, Driggers III WB, De la Parra-Venegas R, Galván-Pastoriza BE, Fox S, Pierce SJ, Dove AD (2017). Long-term assessment of whale shark population demography and connectivity using photo-identification in the Western Atlantic Ocean. PLOS ONE.

[ref-49] McKinney JA, Hoffmayer ER, Wu W, Fulford R, Hendon J (2012). Feeding habitat of the whale shark *Rhincodon typus* in the northern Gulf of Mexico determined using species distribution modelling. Marine Ecology Progress Series.

[ref-50] Meekan M, Bradshaw C, Press M, McLean C, Richards A, Quasnichka S, Taylor J (2006). Population size and structure of whale sharks (*Rhincodon typus*) at Ningaloo Reef Western Australia. Marine Ecology-Progress Series.

[ref-51] Meekan MG, Jarman SN, McLean C, Schultz MB (2009). DNA evidence of whale sharks (*Rhincodon typus*) feeding on red crab (Gecarcoidea natalis) larvae at Christmas Island, Australia. Marine and Freshwater Research.

[ref-52] Mélin F, Hoepffner N, Morales J, Stuart V, Platt T, Sathyendranath S (2011). Monitoring phytoplankton productivity from satellite—an aid to marine resources management. Handbook of satellite remote sensing image interpretation: applications for marine living resources conservation and management.

[ref-53] MEA (2005). Millennium ecosystem assessment, ecosystems and human well-being: biodiversity synthesis.

[ref-54] Motta PJ, Maslanka M, Hueter RE, Davis RL, De la Parra R, Mulvany SL, Habegger ML, Strother JA, Mara KR, Gardiner JM, Tyminski JP (2010). Feeding anatomy, filter-feeding rate, and diet of whale sharks *Rhincodon typus* during surface ram filter feeding off the Yucatan Peninsula, Mexico. Zoology.

[ref-55] NASA (2014). Goddard space flight center, ocean ecology laboratory, ocean biology processing group. http://oceancolor.gsfc.nasa.gov/cgi/l3.

[ref-56] International Union for Conservation of Nature (IUCN) (2005). Rhincodon typus.

[ref-57] Nelson DR, McKibben JN, Strong Jr WR, Lowe CG, Sisneros JA, Schroeder DM, Lavenberg RJ (1997). An acoustic tracking of a megamouth shark, Megachasma pelagios: a crepuscular vertical migrator. Environmental Biology of Fishes.

[ref-58] Norman BM, Whitty JM, Beatty SJ, Reynolds SD, Morgan DL (2017). Do they stay or do they go? Acoustic monitoring of whale sharks at Ningaloo Marine Park, Western Australia. Journal of Fish Biology.

[ref-59] Perry CT (2017). Age and growth of whale sharks (*Rhincodon typus*) near the South Ari Atoll, Maldives.

[ref-60] Pierce SJ, Norman B (2016). Rhincodon typus. http://www.iucnredlist.org/details/19488/0.

[ref-61] Pine R (2007). Donsol whale shark tourism and coastal resource management, a case study on the Philippines [online] WWF-Philippines. http://www.chm.ph/index.php?option=com_docman&task=doc_download&gid=216&Itemid=95.

[ref-62] Platt T, Herman AW (1983). Remote sensing of phytoplankton in the sea: surface-layer chlorophyll as an estimate of water-column chlorophyll and primary production. International Journal of Remote Sensing.

[ref-63] Pravin P (2000). Whale shark in the Indian coast–need for conservation. Current Science.

[ref-64] Prebble CE, Rohner CA, Pierce SJ, Trueman C (2016). Inter-annual feeding ecology of resident whale sharks from Mafia Island, Tanzania.

[ref-65] Quiros A (2007). Whale shark “ecotourism” in the Philippines and Belize: evaluating conservation and community benefits.

[ref-66] Ramírez-Macías D, Saad G (2016). Key elements for managing whale shark tourism in the Gulf of California.

[ref-67] Ramírez-Macías D, Vázquez-Haikini A, Luja V, Murillo R, Mata R (2016). Mapping the path of the biggest fish: the whale shark from the Mexican Pacific side.

[ref-68] Read AJ, Drinker P, Northridge S (2006). Bycatch of marine mammals in US and global fisheries. Conservation Biology.

[ref-69] Reynolds SD, Normans BM, Wilson RP, Bushell H, Neill SO, Morgan DL (2016). Where the whale sharks are: an innovative satellite tagging programme to track the movements of whale sharks from Ningaloo Reef, Western Australia.

[ref-70] Riley M, Hale M, Harman A, Rees R (2010). Analysis of whale shark Rhincondon typus aggregations near South Ari Atoll, Maldives Archipelago. Aquatic Biology.

[ref-71] Robinson DP, Jaidah MY, Bach S, Lee K, Jabado RW, Rohner CA, March A, Caprodossi S, Henderson AC, Mair JM, Ormond R (2016). Population structure, abundance and movement of whale sharks in the Arabian Gulf and the Gulf of Oman. PLOS ONE.

[ref-72] Robinson DP, Jaidah MY, Bach SS, Rohner CA, Jabado RW, Ormond R, Pierce SJ (2017). Some like it hot: repeat migration and residency of whale sharks within an extreme natural environment. PLOS ONE.

[ref-73] Robinson DP, Jaidah MY, Jabado RW, Lee-Brooks K, El-Din NMN, Malki AAA, Elmeer K, McCormick PA, Henderson AC, Pierce SJ, Ormond RF (2013). Whale sharks, *Rhincodon typus*, aggregate around offshore platforms in Qatari waters of the Arabian Gulf to feed on fish spawn. PLOS ONE.

[ref-74] Rohner CA, Armstrong AJ, Pierce SJ, Prebble CE, Cagua EF, Cochran JE, Berumen ML, Richardson AJ (2015). Whale sharks target dense prey patches of sergestid shrimp off Tanzania. Journal of Plankton Research.

[ref-75] Rohner CA, Pierce SJ, Marshall AD, Weeks SJ, Bennett MB, Richardson AJ (2013). Trends in sightings and environmental influences on a coastal aggregation of manta rays and whale sharks. Marine Ecology Progress Series.

[ref-76] Rohner CA, Pierce SJ, Prebbe CE, Igulu M, Kuguru B, Cagua EF, Cochran JE, Berumen ML, Paulsen J, Rubens J (2016). Caught in the net: a small, resident group of whale sharks feeding among fishing boats.

[ref-77] Rohner CA, Richardson AJ, Jaine FR, Bennett MB, Weeks SJ, Cliff G, Robinson DP, Reeve-Arnold KE, Pierce SJ (2018). Satellite tagging highlights the importance of productive Mozambican coastal waters to the ecology and conservation of whale sharks. PeerJ.

[ref-78] Rowat D (2007). Occurrence of whale shark (*Rhincodon typus*) in the Indian Ocean: a case for regional conservation. Fisheries Research.

[ref-79] Rowat D, Brooks KS (2012). A review of the biology, fisheries and conservation of the whale shark *Rhincodon typus*. Journal of Fish Biology.

[ref-80] Rowat D, Gore M (2007). Regional scale horizontal and local scale vertical movements of whale sharks in the Indian Ocean off Seychelles. Fisheries Research.

[ref-81] Rowat D, Meekan MG, Engelhardt U, Pardigon B, Vely M (2007). Aggregations of juvenile whale sharks (*Rhincodon typus*) in the Gulf of Tadjoura, Djibouti. Environmental Biology of Fishes.

[ref-82] Rowat D, Speed CW, Meekan MG, Gore MA, Bradshaw CJ (2009). Population abundance and apparent survival of the vulnerable whale shark *Rhincodon typus* in the Seychelles aggregation. Oryx.

[ref-83] Schmidt JV, Schmidt CL, Ozer F, Ernst RE, Feldheim KA, Ashley MV, Levine M (2009). Low genetic differentiation across three major ocean populations of the whale shark, *Rhincodon typus*. PLOS ONE.

[ref-84] Sequeira AM, Mellin C, Fordham DA, Meekan MG, Bradshaw CJ (2014). Predicting current and future global distributions of whale sharks. Global Change Biology.

[ref-85] Sequeira AMM, Mellin C, Meekan MG, Sims DW, Bradshaw CJA (2013). Inferred global connectivity of whale shark *Rhincodon typus* populations. Journal of Fish Biology.

[ref-86] Sequeira A, Mellin C, Rowat D, Meekan MG, Bradshaw CJ (2012). Ocean-scale prediction of whale shark distribution. Diversity and Distributions.

[ref-87] Sims DW (2008). Sieving a living: a review of the biology, ecology and conservation status of the plankton-feeding basking shark cetorhinus maximus. Advances in Marine Biology.

[ref-88] Sims DW, Southall EJ, Richardson AJ, Reid PC, Metcalfe JD (2003). Seasonal movements and behaviour of basking sharks from archival tagging: no evidence of winter hibernation. Marine Ecology Progress Series.

[ref-89] Sleeman JC, Meekan MG, Wilson SG, Jenner CK, Jenner MN, Boggs GS, Steinberg CC, Bradshaw CJ (2007). Biophysical correlates of relative abundances of marine megafauna at Ningaloo Reef, Western Australia. Marine and Freshwater Research.

[ref-90] Smith ANRI (1828). Descriptions of new, or imperfectly known objects of the animal kingdom, found in the south of Africa. South African Commercial Advertiser.

[ref-91] Springer S (1967). Social organization of shark populations. Sharks, Skates and Rays.

[ref-92] Stewart BS, Wilson SG (2005). Threatened fishes of the world: *Rhincodon typus* (Smith 1828)(Rhincodontidae). Environmental Biology of Fishes.

[ref-93] Sun L, Sinclair-Taylor TH, Cochran JE, Hardenstine RS, Berumen ML (2016). Vertical movement patterns of juvenile whale sharks *Rhincodon typus* at a seasonal aggregation in Saudi Arabian Red Sea.

[ref-94] Tamura R, Kobayashi K, Takano Y, Miyashiro R, Nakata K, Matsui T (2017). Best subset selection for eliminating multicollinearity. Journal of the Operations Research Society of Japan.

[ref-95] Theberge MM, Dearden P (2006). Detecting a decline in whale shark *Rhincodon typus* sightings in the Andaman Sea, Thailand, using ecotourist operator-collected data. Oryx.

[ref-96] Thomson JA, Araujo G, Labaja J, McCoy E, Murray R, Ponzo A (2017). Feeding the world’s largest fish: highly variable whale shark residency patterns at a provisioning site in the Philippines. Royal Society Open Science.

[ref-97] Thums M, Meekan M, Stevens J, Wilson S, Polovina J (2013). Evidence for behavioural thermoregulation by the world’s largest fish. Journal of the Royal Society Interface.

[ref-98] Tyminski JP, De la Parra-Venegas R, Cano JG, Hueter RE (2015). Vertical movements and patterns in diving behavior of whale sharks as revealed by pop-up satellite tags in the eastern Gulf of Mexico. PLOS ONE.

[ref-99] Wilson SG (2004). Basking sharks (Cetorhinus maximus) schooling in the southern Gulf of Maine. Fisheries Oceanography.

[ref-100] Wilson SG, Polovina JJ, Stewart BS, Meekan MG (2006). Movements of whale sharks (*Rhincodon typus*) tagged at Ningaloo Reef, Western Australia. Marine Biology.

[ref-101] Wolanski E, Hamner WM (1988). Topographically controlled fronts in the ocean and their biological influence. Science.

[ref-102] Zavala-Hidalgo J, Gallegos-García A, Martínez-López B, Morey SL, O’Brien JJ (2006). Seasonal upwelling on the western and southern shelves of the Gulf of Mexico. Ocean Dynamics.

[ref-103] Ziegler J, Dearden P, Rollins R (2012). But are tourists satisfied? Importance-performance analysis of the whale shark tourism industry on Isla Holbox, Mexico. Tourism Management.

[ref-104] Zimmerman DW (1987). Comparative power of Student t test and Mann-Whitney U test for unequal sample sizes and variances. The Journal of Experimental Education.

[ref-105] Zuur AF, Ieno EN, Elphick CS (2010). A protocol for data exploration to avoid common statistical problems. Methods in Ecology and Evolution.

